# Spontaneous Vertebral Artery Dissection Is an Under-Recognized Cause of Stroke in Young Patients: Two Case Reports and Review

**DOI:** 10.7759/cureus.9183

**Published:** 2020-07-14

**Authors:** Ahmed Elkady, Sara Ghazal, Abeer Alanzi, Mostafa Madkour, Majid F Bakheet

**Affiliations:** 1 Department of Neurology, Saudi German Hospital, Jeddah, SAU; 2 Department of Neurology, King Abdullah Medical City, Mecca, SAU

**Keywords:** vertebral artery dissection, headache, neck pain, ischemic stroke, neuroimaging

## Abstract

Cervicocerebral artery dissection (CAD) is an important and under-recognized cause of strokes in young and middle-aged patients. Spontaneous vertebral artery dissection (VAD) is a rare condition that can potentially cause a stroke without any preceding trauma or other causes of dissection. VAD rarely simulates classical headache syndromes. In this report, we discuss two young patients who were initially misdiagnosed as cases of headache until they presented with ischemic events, and were eventually diagnosed with spontaneous VAD. Case 1 involves a 41-year-old male patient who presented with severe headache radiating to left posterior neck pain and dizziness. He was initially misdiagnosed as a case of cervicogenic headache. He was subsequently diagnosed with extracranial VAD complicated by a delayed embolic ischemic stroke. However, he made full recovery within the next few days. Case 2 pertains to a 33-year-old female patient who presented with right-sided headache mimicking migraine; later on, new neurological signs prompted a diagnosis of acute ischemic infarction as a complication of intracranial VAD. In conclusion, VAD should be seriously considered when dealing with patients complaining of the first attack of headache that mimics migraine or those with cervicogenic headaches, which fail to respond to the usual treatment. Moreover, posterior circulation stroke among young patients or stroke with pain in the head and neck should be investigated carefully with extensive neuroimaging. Finally, prompt and accurate diagnosis of VAD followed by proper treatment is crucial for good outcomes and will prevent disability or even fatal complications in patients.

## Introduction

The incidence of stroke among patients aged >65 years has decreased over the past couple of decades; however, population-based studies have shown a growing incidence of ischemic strokes in young adults [1]. Cervicocerebral artery dissection (CAD) is a definite etiology for stroke among young patients. CAD is further classified into internal carotid artery dissection (ICAD) and vertebral artery dissection (VAD) [2,3]. Although CAD is usually associated with trauma, spontaneous dissection is being increasingly reported with the advent of advanced neurovascular imaging [4]. While both ICAD and VAD are associated with headache and neck pain and delayed ischemic events, ICAD could be recognized earlier due to its presentation with focal neurological signs as Horner syndrome [5]. VAD is usually misinterpreted as a musculoskeletal disease in its early presentation; yet, early and prompt diagnosis of this condition could prevent significant disability or even life-threatening stroke [6]. The outcome of CAD is unpredictable. While some patients achieve a full recovery, some develop permanent neurologic deficits, and the condition can be fatal in a small number of patients.

The vague presentation and unpredicted outcomes associated with the condition have motivated the authors to present two cases of middle-aged patients, who presented with complaints of severe headache and neck pain. Initially, both cases were misdiagnosed: patient 1 was treated as a case of cervicogenic headache, and patient 2 was misdiagnosed as a case of migraine with aura. However, ischemic events were eventually encountered in both cases, and subsequent neuroimaging revealed spontaneous VAD.

## Case presentation

Case 1

**Figure 1:**
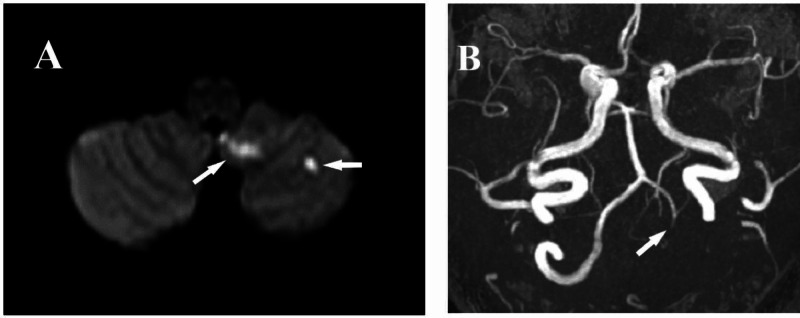
MRI and MRA findings – case 1 (A) Brain MRI, DWI sequence showing multiple restricted diffusion lesions in the left cerebellar region (arrows). (B) Brain MRA showing that only the V4 segment is visualized, while V1-V3 segments are absent (arrow) MRI: magnetic resonance imaging; DWI: diffusion-weighted imaging; MRA: magnetic resonance angiography

A 41-year-old ex-smoker male patient with no past medical history presented to the ED. He complained of a headache of subacute onset radiating to the left side of the neck, dizziness, and nausea. His symptoms had started two weeks ago upon waking up from sleep and then worsened during his daily activities. He had received analgesics that had improved his symptoms temporarily. On examination, the patient was vitally stable, afebrile, and he rated his headache as a 6 on a scale of 1-10. Although his dizziness did not interfere with his ability to walk, it increased with motion or when he changed his position and caused him to vomit during the evaluation. His neurological examination was unremarkable for any focal neurological signs except for neck muscle spasm and tenderness. His cervical X-ray showed misalignment between C3-C4 with narrowing disc space. Muscle relaxants, analgesics, and antiemetic were prescribed. An orthopedic consultation was advised. One week later, he presented again with a progressive course of vertigo, headache, and frequent nausea and vomiting. Additionally, he reported numbness of fingers and toes and pain around the eyes. Neurological evaluation revealed left-side ataxia during finger to nose and heel to shin test, without gait ataxia. Magnetic resonance imaging (MRI) stroke protocol showed recent multiple recent ischemic infarctions in the left cerebellum (Figure 1A). Magnetic resonance angiography (MRA) showed good cerebral vasculature; however, the left vertebral artery was not visualized except for the V4 segment (Figure 1B).

**Figure 2:**
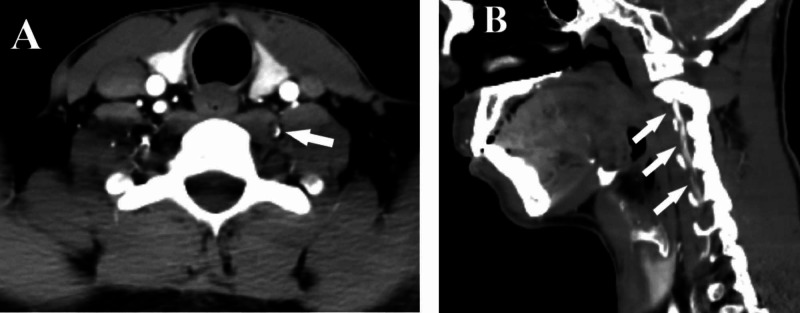
CTA findings – case 1 CTA of cervical arteries showing left vertebral artery intramural hematoma looking crescent-shaped (arrow) in the axial view (A), while it looks spiral (arrows) along (V1-V3) segments in the sagittal reconstructed view (B) CTA: computed tomography angiography

CT angiography (CTA) of the brain and neck with intravenous contrast was performed, and it showed crescent-shaped intramural hematoma inside the left vertebral artery in the axial view (Figure 2A), while in CTA reconstruction sagittal images, it looked spiral within a long segment of 6.5 cm (V1-V3) (Figure 2B), which is characteristic of vessel wall dissection.

**Figure 3:**
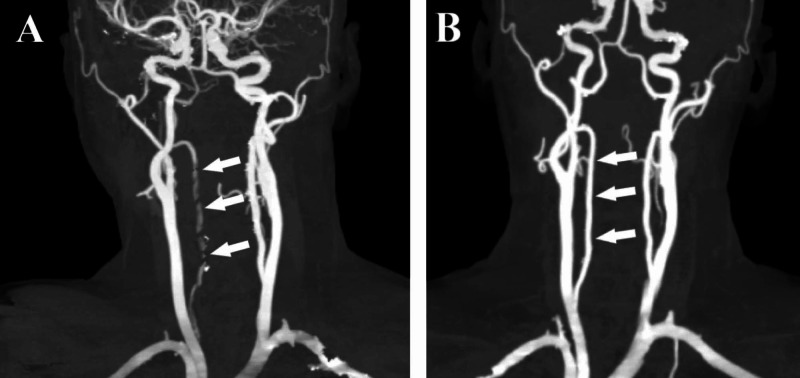
3D maximum-intensity CTA on admission and follow-up CTA three months after discharge – case 1 (A) Three-dimensional maximum-intensity CTA of left vertebral artery on admission showing tapered stenosis (string sign) within V1–V3 segments (arrows). (B) Follow-up CTA after three months showing complete resolution of tapered flow (arrows) CTA: computed tomography angiography

Moreover, three-dimensional (3D) CTA images revealed corresponding tapered stenosis from its origin until the C2 vertebrae level, giving string sign appearance (Figure 3A). A diagnosis of spontaneous VAD was made; dual antiplatelets were started for symptomatic treatment for vertigo and vomiting. This led to an improvement of symptoms over the next few days, and the patient was discharged home. CTA was repeated after three months, which showed a near-complete resolution of the arterial dissection in the involved segments (Figure 3B).

Case 2

**Figure 4:**
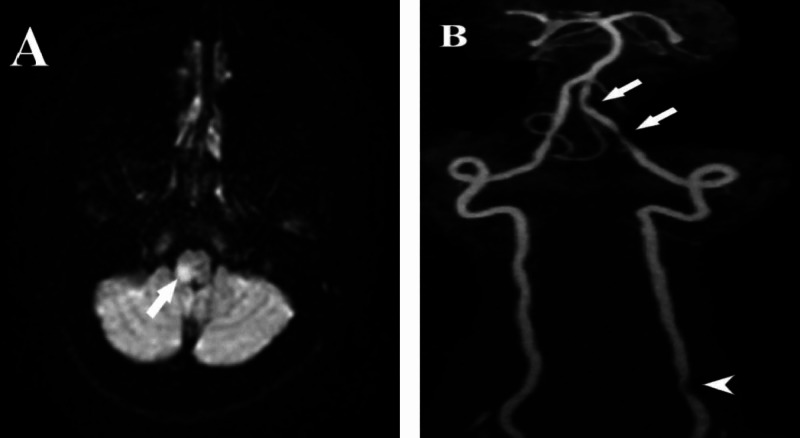
MRI and MRA findings – case 2 (A) Brain MRI, DWI sequence showing restricted diffusion within the right lateral medulla (arrow). (B) Brain MRA showing multiple focal stenotic lesions in the right vertebral artery (V4) segment (arrows), and (V1) segment (head arrow) MRI: magnetic resonance imaging; DWI: diffusion-weighted imaging; MRA: magnetic resonance angiography

A 33-year-old woman presented to the ED with a severe, right-sided, throbbing headache for the last five days, which was associated with vomiting and photophobia. She denied any trauma or manipulation except for her daily stretches. She had a medical history of catamenial migraine without aura, mostly pulsatile, and localized to the frontotemporal region. She reported that her presenting attack had been preceded by a sudden onset of visual symptoms in the left visual field and characterized by several scotomas, zig-zag lines, and dark holes. Moreover, she rated her pain intensity as 7 out of 10 and described an unusual location in an occipital region with mild right neck tenderness. She reported some response to acetazolamide with analgesics and triptans in severe attacks. MRI brain and MR venography were unremarkable; hence, she was treated as status migrainosus with intravenous (IV) valproic acid and metoclopramide with partial improvement. Three days later, she presented again to the ED with frequent vomiting, hiccups, dizziness, dysarthria, and blurring of vision. Neurological examination revealed the loss of sensation over the right side of the face, right upper limb ataxia, nystagmus with right eye ptosis, and constricted pupil (Horner syndrome). A new MRI of the brain showed right lateral medullary infarction (Figure 4A), and MRA showed focal stenosis within V4 segments of the right vertebral artery (Figure 4B).

**Figure 5:**
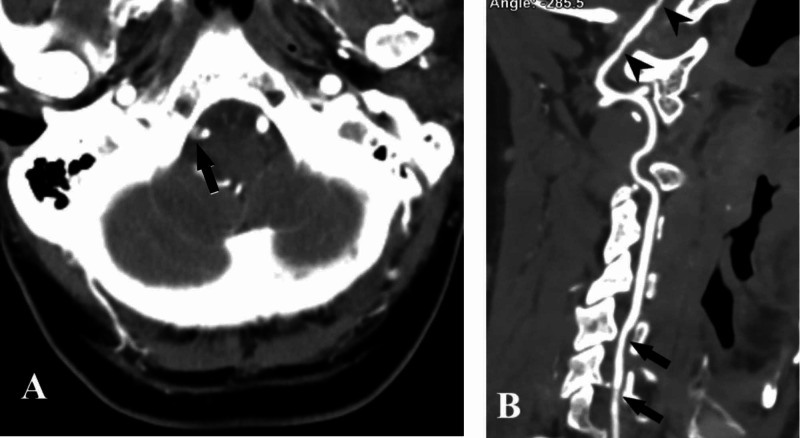
CTA findings – case 2 CTA of the right vertebral artery showing a double-lumen sign in (V4) segment (arrow) in the axial view, while sagittal reconstructed view (B) shows multiple stenotic lesions in V1 (head arrows) and in V1-V2 (arrows) CTA: computed tomography angiography

CTA was performed, and it revealed a double-lumen of the right vertebral artery in the axial view (Figure 5A). CTA reconstruction sagittal images showed multiple stenotic lesions within the V1 and V4 of the right vertebral artery (Figure 5B). While a double-lumen sign in the V4 segment is highly suggestive of dissection, multiple stenotic lesions warrant more work-up to exclude other etiologies like reversible cerebral vasoconstriction syndrome (RCVS) and vasculitis.

**Figure 6:**
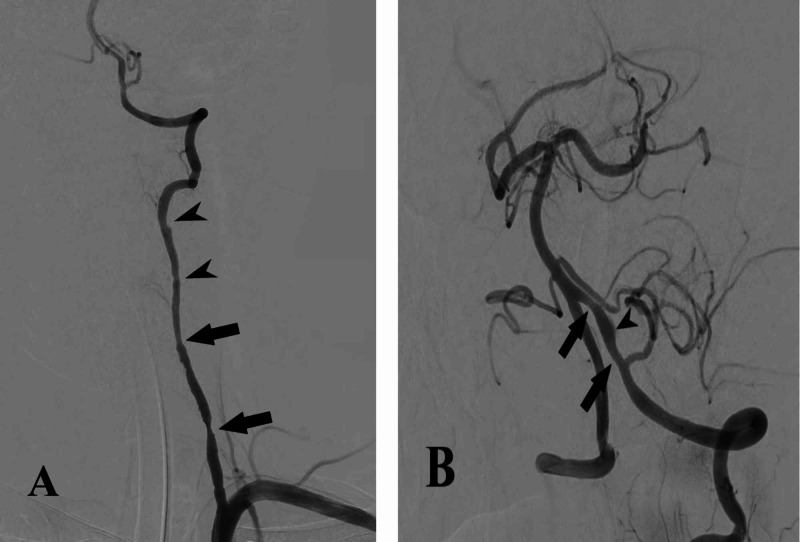
Cerebral conventional angiography findings – case 2 Cerebral conventional angiography of right vertebral artery; cervical part (A) showing bead-like stenosis of V1 segment (arrows) followed by heterogeneous irregularities of the lumen (head arrows) (pearl and string sign); intracranial part (B) revealed another tapered stenosis after the branching of the posterior inferior cerebellar artery (arrows), with distal dilatation and intimal flap rising (head arrow)

Conventional cerebral angiography was performed, which revealed bead-like stenosis of the V1 segment of the right vertebral artery with distal dilatation and heterogeneous irregularities of the lumen (Figure 6A), which was evaluated as a pearl and string sign. Moreover, the intracranial part revealed another tapered stenosis after the branching of the posterior inferior cerebellar artery (PICA) with distal dilatation and heterogeneous opacification of the lumen (Figure 6B).

A diagnosis of spontaneous VAD was made. Treatment with oral anticoagulant was started together with symptomatic treatment for other symptoms with improvement, except for hiccups, which continued for another two weeks after discharge from the hospital. Because of her good recovery from all symptoms, she refused to undergo a follow-up CTA three months later.

## Discussion

Spontaneous CAD is a common cause of stroke in young adults, with a prevalence of up to 20% in patients who are <50 years old. It has a variable incidence rate as many cases may go undiagnosed due to minor self-limited clinical symptoms in some people, while others will not have access to medical facilities [7]. Spontaneous VAD is a common cause of posterior circulation ischemic stroke in young adults. Headache and pain occur as the most common, initial, and even isolated symptoms in VAD [4]. We discussed two cases with spontaneous VAD, which were initially misdiagnosed as cervicogenic headache (case 1) and as migraine with aura (case 2), respectively. The accurate diagnosis was delayed in both cases until ischemic events occurred. Minor trauma preceding spontaneous VAD is reported in many patients, and this includes chiropractic manipulation, various sporting activity-related injuries like a whiplash injury, and injuries caused by stretching and sudden neck movements (case 2) and severe coughing [6]. Furthermore, genetic connective tissue conditions, migraine with aura, hypertension, hyperhomocysteinemia, and a history of recent infections have been reported more in patients with VAD than in healthy controls [7]. The exact pathophysiology of CAD is poorly understood; however, dissection usually starts with a tear in the intima or rupture of the vasa vasorum with bleeding within the media resulting in separation of the vessel wall layers and a false lumen. Moreover, a hematoma can expand towards the adventitia causing a sub-adventitial dissection, which may be accompanied by a dissecting pseudoaneurysm, or towards the intima resulting in a subintimal dissection causing luminal narrowing [8]. Spontaneous VAD typically begins with ipsilateral neck pain or headache, while ischemic events with focal neurological signs could be delayed for days or even weeks. The International Headache Society (IHS) has proposed a list of criteria to differentiate CAD headache from other, less serious, types of headaches such as cervicogenic (case 1), or migraine with aura (case 2) (Table 1) [9].

**Table 1:** The International Headache Society (IHS) criteria for the diagnosis of headache or facial or neck pain attributed to cervical carotid or vertebral artery dissection ICHD-3: The International Classification of Headache Disorders 3rd edition

ICHD-3 criteria
A. Cervical carotid or vertebral dissection has been diagnosed with the fulfillment of criteria B and C
B. Evidence of causation demonstrated by at least two of the following: 1. pain has developed in close temporal relation to other local signs of the cervical artery dissection or has led to its diagnosis. 2. Either or both of the following: a) pain has significantly worsened in parallel with other signs of the cervical artery dissection; b) pain has significantly improved or resolved within one month of its onset. 3. Either or both of the following: a) pain is severe and continues for days or longer; b) pain precedes signs of acute retinal and/or cerebral ischemia. 4. Pain is unilateral and ipsilateral to the affected cervical artery
C. Either of the following: 1: headache has resolved within three months; 2. headache has not yet resolved but three months have not yet passed
D. Not better accounted for by another ICHD-3 diagnosis

Other clinical manifestations of extracranial VAD are usually attributed to ischemic stroke (cases 1 and 2). These include, but are not limited to, dizziness, nausea, vomiting, double vision, vertigo, ataxia, and dysarthria. Cerebellar infarction and lateral medullary (Wallenberg syndrome) are the most common types of strokes (cases 1 and 2 respectively) [10]. The clinical presentations of intracranial VAD are similar to extracranial symptoms, but usually associated with more severe neurological deficits; moreover, about half of intracranial VAD cases are associated with thunderclap headache due to subarachnoid hemorrhage (SAH), which results from a ruptured dissected vessel or pseudoaneurysm [11]. Although the exact mechanism of stroke in patients with VAD is uncertain, artery-to-artery embolism of a thrombus within a false lumen is believed to be the most common mechanism in extracranial VAD (case 1) [7]. Conversely, ischemic stroke related to intracranial VAD could be a consequence of in situ occlusions of a branch of the dissected vessel [11]. Typical radiological features can be visualized with MRI, MRA, and CTA (case 1) as they are sensitive to detect intramural hematoma; however, some cases, especially intracranial VAD, will need confirmation by conventional angiography (case 2), if noninvasive techniques were not conclusive [12]. Recently, high-resolution MRI (HR-MRI) has been reported to offer the advantage of direct visualization both of vessel wall abnormalities and an intraluminal thrombus; however, most patients do not have access to such techniques [13].

The main purpose of treating VAD is either to stabilize the thrombus or to reduce further thrombus formation, which could primarily prevent stroke; However, if a stroke has already occurred, the aim is to prevent progression or recurrence [14]. So far, only the Cervical Artery Dissection in Stroke Study (CADISS) trial has assessed the use of antiplatelets versus anticoagulants to reduce stroke risk; they concluded that there was no difference between treatment groups either in outcome events or the rate of recanalization [15]. The treatment of intracranial VAD is much more complicated and warrants optimized management strategies. Firstly, we should exclude SAH; it should be followed by the assessment of any wall malformation that could require endovascular intervention. Finally, we can proceed to use either antiplatelets or anticoagulants [11].

## Conclusions

Spontaneous VAD should be highly suspected in patients who present with the first attack of severe cervicogenic headache, especially those with a history of minor trauma, and in patients presenting with the first attack of headache with visual disturbances that mimic migraine with aura. Furthermore, prolonged unilateral headache not responding to standard medications should be evaluated by extensive neuroimaging. This will help in early diagnosis of VAD and timely initiation of appropriate therapy for preventing stroke.
